# Concordance of a decision algorithm and multidisciplinary team meetings for patients with liver cancer—a study protocol for a randomized controlled trial

**DOI:** 10.1186/s13063-023-07610-8

**Published:** 2023-09-09

**Authors:** Sharlyn S. T. Ng, Robert Oehring, Nikitha Ramasetti, Roland Roller, Philippe Thomas, Yuxuan Chen, Simon Moosburner, Axel Winter, Max-Magnus Maurer, Timo A. Auer, Can Kamali, Johann Pratschke, Christian Benzing, Felix Krenzien

**Affiliations:** 1 Department of Surgery, Charité – Universitätsmedizin Berlin, corporate member of Freie Universität Berlin and Humboldt-Universität zu Berlin, Augustenburger Platz 1, 13353 Berlin, Germany; 2 German Research Center for Artificial Intelligence (DFKI), Berlin, Germany; 3 Berlin Institute of Health at Charité – Universitätsmedizin Berlin, Charitéplatz 1, 10117 Berlin, Germany; 4 Department of Radiology, Charité – Universitätsmedizin Berlin, corporate member of Freie Universität Berlin, Humboldt-Universität zu Berlin, Augustenburger Platz 1, 13353 Berlin, Germany

**Keywords:** Decision support systems, Clinical, Tumor board, Multidisciplinary team meeting, Carcinoma, Hepatocellular, Cholangiocarcinoma, Artificial intelligence, Machine learning, Natural language processing

## Abstract

**Introduction:**

Multidisciplinary team meetings (MDMs), also known as tumor conferences, are a cornerstone of cancer treatments. However, barriers such as incomplete patient information or logistical challenges can postpone tumor board decisions and delay patient treatment, potentially affecting clinical outcomes. Therapeutic Assistance and Decision algorithms for hepatobiliary tumor Boards (ADBoard) aims to reduce this delay by providing automated data extraction and high-quality, evidence-based treatment recommendations.

**Methods and analysis:**

With the help of natural language processing, relevant patient information will be automatically extracted from electronic medical records and used to complete a classic tumor conference protocol. A machine learning model is trained on retrospective MDM data and clinical guidelines to recommend treatment options for patients in our inclusion criteria. Study participants will be randomized to either MDM with ADBoard (Arm A: MDM-AB) or conventional MDM (Arm B: MDM-C). The concordance of recommendations of both groups will be compared using interrater reliability. We hypothesize that the therapy recommendations of ADBoard would be in high agreement with those of the MDM-C, with a Cohen’s kappa value of ≥ 0.75. Furthermore, our secondary hypotheses state that the completeness of patient information presented in MDM is higher when using ADBoard than without, and the explainability of tumor board protocols in MDM-AB is higher compared to MDM-C as measured by the System Causability Scale.

**Discussion:**

The implementation of ADBoard aims to improve the quality and completeness of the data required for MDM decision-making and to propose therapeutic recommendations that consider current medical evidence and guidelines in a transparent and reproducible manner.

**Ethics and dissemination:**

The project was approved by the Ethics Committee of the Charité – Universitätsmedizin Berlin.

**Registration details:**

The study was registered on ClinicalTrials.gov (trial identifying number: NCT05681949; https://clinicaltrials.gov/study/NCT05681949) on 12 January 2023.

**Supplementary Information:**

The online version contains supplementary material available at 10.1186/s13063-023-07610-8.

## World Health Organization Trial Registration Data Set

**Table:** 

Data category	Information
Primary registry and trial identifying number	ClinicalTrials.gov (NCT05681949) https://clinicaltrials.gov/study/NCT05681949
Date of registration in primary registry	12 January 2023
Source of monetary or material support	Federal Joint Committee of Germany (*Gemeinsamer Bundesausschuss; Innovationsfonds*) grant number 01VSF21047
Primary sponsor	Charité-Universitätsmedizin Berlin
Contact for public queries	Felix Krenzien, MD, Department of Surgery, Campus Charité Mitte and Campus Virchow-Klinikum, Charité – Universitätsmedizin Berlin, Augustenburger Platz 1, 13353 Berlin, Germany. Email: felix.krenzien@charite.de; Phone: (+ 49) 30 450 652 006; Fax: (+ 49) 30 450 552 900
Contact for scientific queries	Felix Krenzien, MD, Department of Surgery, Campus Charité Mitte and Campus Virchow-Klinikum, Charité – Universitätsmedizin Berlin, Augustenburger Platz 1, 13353 Berlin, Germany. Email: felix.krenzien@charite.de; Phone: (+ 49) 30 450 652 006; Fax: (+ 49) 30 450 552 900
Public title	Therapeutic Assistance and Decision Algorithms for Hepatobiliary Tumor Boards
Scientific title	Evaluation of the Trustworthiness of the Application of Artificial Intelligence and Decision Support Systems for the Creation of Tumor Conference Protocols
Country of recruitment	Germany
Health conditions or problems studied	Primary and secondary liver tumors
Interventions	No interventions
Key inclusion and exclusion criteria	Inclusion criteria are (1) patients above 18 years of age; (2) valid informed consent; (3) patient information available in the hospital information system and Health Data Platform; (4) registration in the hepatobiliary MDM; (5) diagnosis of any of the following: (a) HCC, mixed cell carcinoma, or fibrolamellar carcinoma; (b) perihilar cholangiocarcinoma; (c) intrahepatic cholangiocarcinoma; (d) colorectal liver metastases; (e) gallbladder carcinoma. Exclusion criteria are (1) patient does not consent or is incapable of giving consent; (2) patient data is unavailable in the hospital information system; (3) patient is seeking for a second opinion and not being treated at the study institution.
Study type	Monocentric, prospective, parallel randomized control trial with a non-inferiority design
Date of first enrollment	Not yet recruiting
Target sample size	1200
Recruitment status	Not yet recruiting
Primary outcomes	Concordance of recommendations of MDM-C and MDM-AB; interrater reliability of the agreement between recommendations of ADBoard and MDM-C; reproducibility of the therapy recommendations made by ADBoard
Key secondary outcomes	Completeness of decision-relevant parameters MDM protocol; explainability of ADBoard measured using the System Causability Scale (SCS) [1]

### Protocol version

Protocol version 2, dated 25 August 2023.

## Introduction

Multidisciplinary team meetings (MDMs) are a crucial component of cancer treatment [2]. MDMs are hosted regularly, usually weekly, and attended by members from various specialties, including oncologists, surgeons, radiation therapists, pathologists, and radiologists. With their combined expertise, they review patients’ medical history and examination findings, and determine the most suitable treatment plan by consensus.

Globally, primary liver cancer is the fourth leading cause of cancer-related death, with nearly 841,000 new cases and 782,000 deaths annually [3]. Demographic changes have led to an increase in older patients with hepatocellular carcinoma (HCC). The European clinical guidelines for the management of HCC strongly recommend discussion at an MDM before determining treatment interventions [3]. A multidisciplinary approach to caring for HCC has been shown to improve patient outcomes [4]. However, there are several barriers to truly effective MDMs, such as the failure of clinicians to submit adequate information in time for the MDM, physicians’ lack of personal knowledge of the patient being discussed, and time and workload pressures [5, 6].

Having a complete set of patient information at the time of the MDM is indispensable for effective decision-making [5, 6]. In the current state, simple online forms or “tumor protocols” such as the Gießen Tumor Documentation System (GTDS) [7] in Germany are used by residents to manually collect relevant patient information and parameters in preparation for the MDM. However, due to heavy clinical workloads, residents are often unable to fill out the form completely prior to the MDM. Finding information in the hospital information system and determining its relevance is time-consuming, especially for inexperienced residents. They often encounter logistical challenges in retrieving patient information [8]; for example, missing medical reports, insufficient patient history, and lack of relevant details on referral forms. Without all the necessary data, treatment decisions are either made on incomplete data or have to be postponed; consequently, the affected patients suffer delays in treatment [5, 9]. Therefore, there is potential for MDM processes to be improved, which can in turn have a significant impact on treatment decision-making and patient outcomes.

ADBoard (Therapeutic Assistance and Decision algorithms for hepatobiliary tumor Boards) aims to improve the MDM process for patients with liver cancer by using different artificial intelligence (AI) methods from the fields of natural language processing (NLP) and machine learning (ML). First, clinical text documents will be processed to automatically complete all required patient information in a conventional tumor protocol. Next, an ML model trained on MDM data will make therapeutic recommendations for specific clinical cases. The algorithm is intended for physician use only: to relieve their administrative workload of manual information collection and documentation, and to provide therapeutic recommendations.

The primary objective of this study is to assess the concordance of conventional MDM (Arm B: MDM-C) recommendations with ADBoard-supported MDM (Arm A: MDM-AB) recommendations. The reproducibility of ADBoard recommendations will be tested. The secondary objectives are (1) to examine whether ADBoard will have and use a more complete set of oncological and patient-specific parameters than is presented at MDM-C and (2) to compare the explainability of MDM-AB recommendations against that of MDM-C.

## Methods and analysis

### Study setting

The single-center study will be conducted at an academic hospital, Campus Virchow-Klinikum, Charité – Universitätsmedizin Berlin, Berlin, Germany.

### Recruitment

All patients enrolled in hepatobiliary MDMs with the diagnoses specified in the inclusion criteria will be invited to join the study.

### Eligibility criteria

#### Inclusion criteria

The inclusion criteria are (1) patients above 18 years of age; (2) valid informed consent; (3) patient information available in the hospital information system and Health Data Platform; (4) registration in the hepatobiliary MDM; and (5) diagnosis of any of the following: (a) HCC, mixed cell carcinoma, or fibrolamellar carcinoma; (b) perihilar cholangiocarcinoma; (c) intrahepatic cholangiocarcinoma; (d) colorectal liver metastases; or (e) gallbladder carcinoma.

#### Exclusion criteria

The exclusion criteria are as follows: (1) patient does not consent or is incapable of giving consent; (2) patient data is unavailable in the hospital information system; and (3) patient is seeking for a second opinion and is not being treated at the study institution.

### Study design

**Fig. 1:**
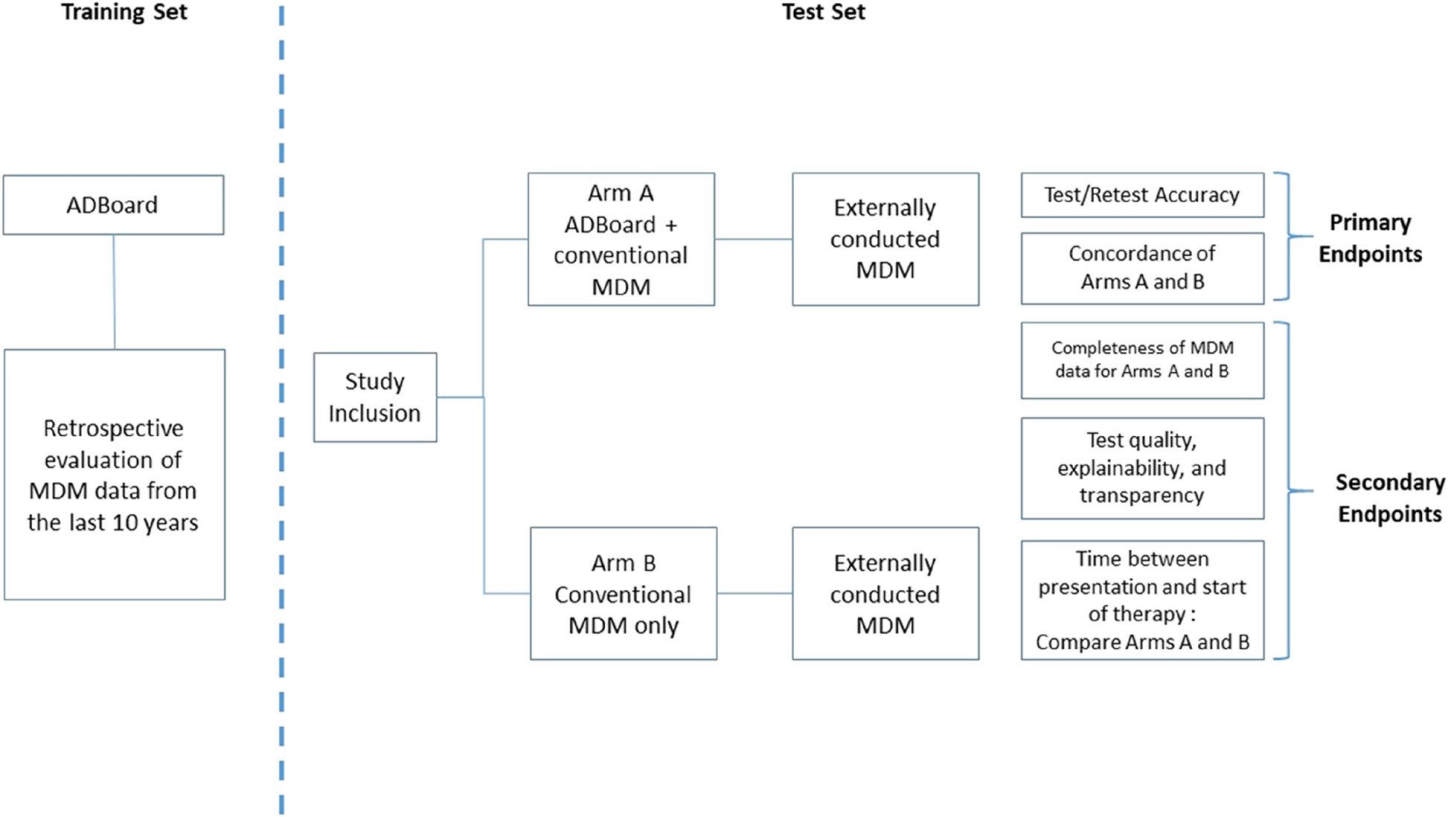
Timeline of the study with primary and secondary endpoints

ADBoard is a monocentric, prospective, parallel randomized controlled trial with a non-inferiority design. Participants will be randomized 1:1 using the randomization module in the Research Electronic Data Capture (REDCap) software [10] into one of two groups: either (a) MDM with ADBoard (MDM-AB) or (b) conventional MDM without ADBoard (MDM-C). Figure 1 shows the timeline of the study with its primary and secondary endpoints.

In the preparation stage, the ML algorithm will be trained using retrospective data of hepatobiliary MDMs. The algorithm will be validated by comparing its recommendation with the conclusions of past MDMs. The recruited cases will then be analyzed prospectively for 30 months using the validated algorithm. They will then be reviewed according to the specified outcome measures.

The basis of ADBoard is the automated systematic collection of patient data necessary for presentation and decision-making in MDMs. Patient data for MDM-AB are automatically deidentified and extracted to a locally hosted database using an application in the internal Health Data Platform. NR ensures data quality by verifying it as it is acquired. Data in the control arm would be manually entered in the GTDS by the physician in charge of the patient’s care, according to conventional practice of the MDM-C. Specifically, the following parameters are recorded, depending on the availability: age; sex; concomitant diseases; liver function; medications; radiological staging (computed tomography, magnetic resonance imaging, X-ray, and/or sonography reports); tumor characteristics such as diameter, extension or positioning relative to vascular structures; pathology findings; and tumor therapies the patient already received.

The synthesis of tumor-specific characteristics and parameters should allow an assignment into the following therapeutic categories: (a) a therapy recommendation according to the current guidelines; (b) if the case is not covered by the guidelines, this should be recognized, and possible therapeutic approaches according to current studies should be suggested; or (c) patient cases that require an individualized decision due to complexity (e.g., relapse with complex disease history or severe concomitant diseases) should be identified and discussed at a conventional MDM.

The follow-up ends with the initiation of the MDM recommendation. Both study arms will be validated by an external, conventional MDM conducted at the end of the follow-up period to serve as a reference. This reference MDM will be conducted by experts in hepatobiliary tumors from the departments of surgery and oncology as soon as the recommendation has been initiated, typically within 4 weeks after conducting MDM-C and MDM-AB. It would only be conducted as part of the study. Its slightly delayed timing would ensure that any information not available at the time of the initial MDM-AB and MDM-C could be processed.

To improve adherence to the study, the research team will ensure the availability of logistics such as the designated computer enabled to run the algorithm and physician availability and presence during the MDM to carry out the intervention.

Both groups of participants would receive baseline care, that is, all cases would be evaluated at a conventional MDM. Participants retain the right to accept or refuse recommended treatments.

### Allocation and blinding

Allocation concealment is ensured as randomization occurs only after participants have been recruited into the trial. A physician (RO) on the research team who is not involved in the decision process of MDMs will generate the allocation sequence, enroll participants, and assign the participants to interventions. Participants will be blinded after assignment to interventions. The data analysts will be blinded to the assignment throughout the study.

### Data collection plan

All participants’ cases would be discussed at MDM-C. For participants randomized to MDM-AB, these cases would be processed by the ADBoard algorithm in real-time, immediately after the MDM-C session has taken place. The outcomes of both MDM-C and MDM-AB would be recorded using secure, web-based REDCap tools hosted at Charité – Universitätsmedizin Berlin [10, 11].

### Outcomes

#### Primary outcome measures

We will study the concordance of the recommendations of MDM-C and MDM-AB. The interrater reliability of the agreement between recommendations of ADBoard and MDM-C will be measured using Cohen’s kappa value, with a target value of ≥ 0.75.

We will also assess the reproducibility of the therapy recommendations made by ADBoard. The intrarater reliability will be measured by testing all participants’ cases several times by ADBoard according to the required sample size with sufficient statistical power (test–retest). Interrater and intrarater reliability will be evaluated descriptively (percentage of agreement, contingency tables), and finally, Cohen’s kappa value will be assessed.

#### Secondary outcome measures

The first secondary outcome measure is the completeness of decision-relevant parameters. To prefill the required parameters in the online tumor protocol, ADBoard searches the health information system for each required parameter. The three possible documentation statuses are (1) “present”—search performed and data are present, (2) “detected as missing”—search performed and data are absent, and (3) “missing”—search was not performed for the required parameter. The outcome is achieved if documentation is complete, defined as documentation status “present” or “detected as missing,” in ≥ 75% of all ADBoard-assisted decisions.

The second secondary outcome measure is the explainability of ADBoard measured using the System Causability Scale (SCS) [1]. The SCS consists of 10 questions. An overall score between 0.2 (lowest quality) and 1.0 (highest quality) can be achieved. Three specialist physicians at the departments of surgery and oncology will complete it, and the mean will be calculated. Achieving a mean score of ≥ 0.8 is considered to have met this outcome.

### Participant timeline

**Fig. 2:**
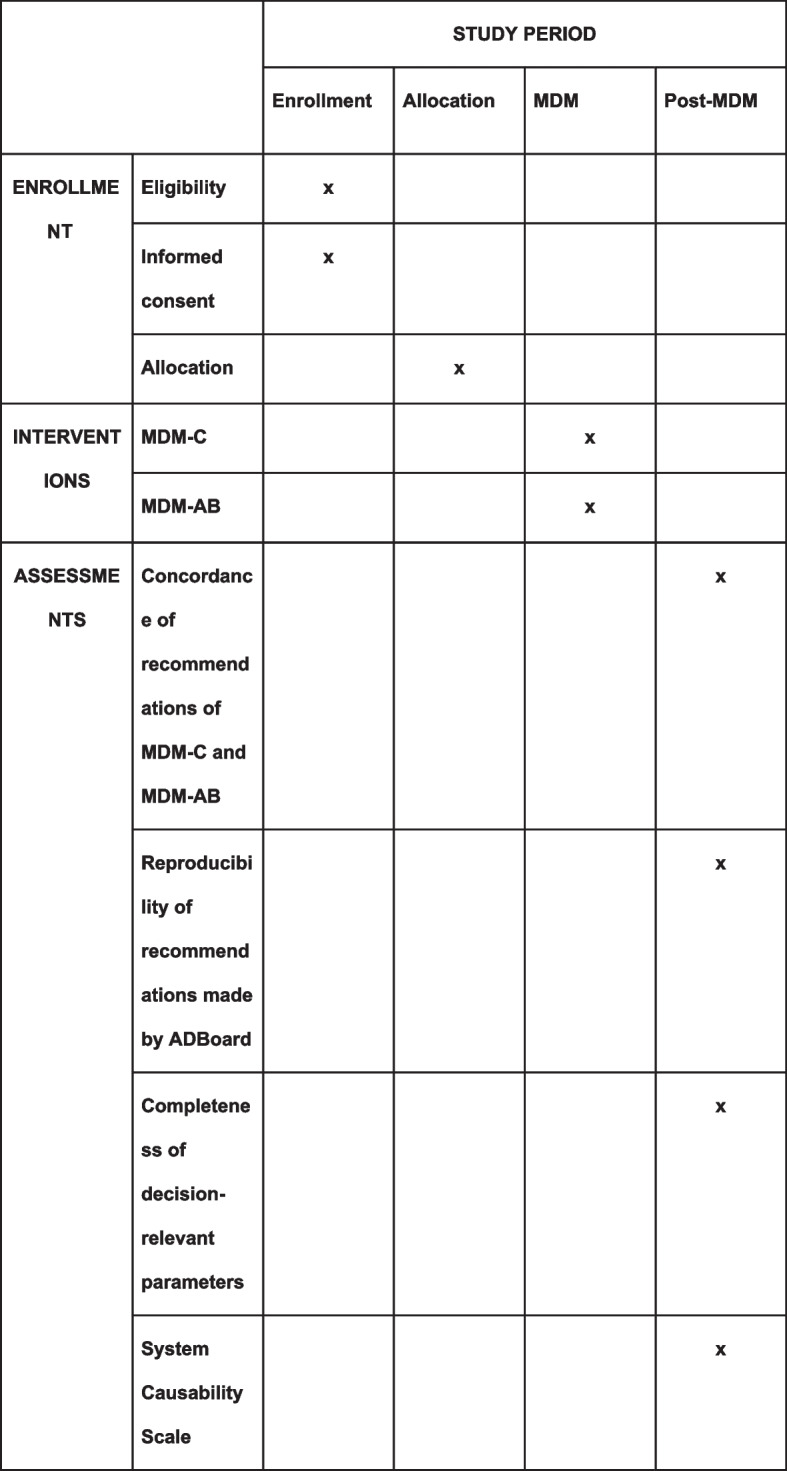
Schedule of enrollment, interventions, and assessments

The schedule of enrollment, interventions, and assessments is displayed below in Fig. 2.

### Harms

We do not foresee any potential harms or adverse effects occurring because of the study. The participants would ultimately receive the same recommendations and treatment options with or without the interventions. The recommendations made by the decision algorithm would be recorded purely for study purposes only.

### Sample size

#### Calculation of sample size

The evaluation of the primary outcome is based on Cohen’s kappa test (inter- and intrarater comparison) for nondichotomous decision-making. There are five possible recommendation categories: surgical resection or transplantation, chemo-/immunotherapy, interventional therapy, re-intervention, and further diagnosis. A five-category nominal scale will be used between different raters or different ratings of the same instance. The frequency of each category is not evenly distributed here but is estimated to be 0.3 for resection or transplantation, 0.3 for chemotherapy, 0.2 for follow-up, 0.1 for intervention, and 0.1 for further diagnosis. To capture any deviation from this estimate, different expressions of the frequencies were included in Table 1. The null hypothesis was defined as a Cohen’s kappa value of 0.7, as reliability below this value can be considered clinically unacceptable. The reliability strength to be achieved was set at 0.8 (primary endpoint: kappa value ≥ 0.75). Based on these specifications, the required numbers of cases were taken from the calculations of Bujang and Adnan [12]. A total of 3000 patients will be included in the retrospective part of the study and 1200 patients will be included in the prospective part.

**Table 1:** Indication of the numbers of cases required for different relative frequencies of a 5-category nominal scale [12]. Number of categories: 5, type I error (*ɑ*): 0.05, null hypothesis (Cohen’s kappa): 0.7, primary endpoint (Cohen’s kappa): 0.8

Relative frequencies	Required number of cases (power 80%)	Required number of cases (power 90%)
0.3, 0.3, 0.2, 0.1, 0.1	240	311
0.3, 0.25, 0.2, 0.1, 0.05	254	329
0.6, 0.1, 0.1, 0.1, 0.1	306	400

The required numbers of cases depending on the relative frequencies of the recommendation types are displayed in Table 1.

### Statistical analysis

#### Planned analysis

To assess the agreement between the MDM-C and MDM-AB recommendations, interrater reliability will be examined.

To examine the reproducibility of ADBoard decisions, intrarater reliability will be studied. A third reference MDM will be conducted externally in the event of discordance between the recommendations of MDM-AB and MDM-C. The interrater reliability between the reference MDM and MDM-AB as well as between the reference MDM and MDM-C will be determined. A Cohen’s kappa value of ≥ 0.75 (*p* < 0.05, *Z* test) is postulated for the interrater reliability for both comparisons. If there is no agreement between MDM-AB and MDM-C, the reference MDM can evaluate which conference is most likely to be guideline- and evidence-based. This arbitrarily chosen value corresponds to a “good to excellent reliability” [13]. The basis of the data analysis is the dataset collected up to T1 (time of the conventional MDM).

Oncological and patient-specific parameters, which are necessary for the therapy decision of the respective disease and are predefined for each tumor type, will be compared between the ADBoard-recommended protocol (based on the dataset collected up to T1), the MDM-C, and the reference MDM (descriptive evaluation, target value for feature expression of the ADBoard decision 100%, *p* < 0.05, Mann–Whitney *U* test). We predict an improvement in the documentation according to the legal requirements.

### System architecture of ADBoard

ADBoard consists of two main technical components: (a) the automatic completion of missing patient information and (b) the automatic treatment recommendation. The purpose and technical implementation of both components are described below.

Structured patient data in tumor protocols can be incomplete, and missing information is often spread across various departments. Examples of missing information are laboratory, radiology, or pathology results, and tumor size or patient status. In the current state, required data must be retrieved individually in a manual and time-consuming fashion. With ADBoard, information (if present) will be automatically extracted using NLP. The process begins with an existing information extraction model, mEx (medical information Extraction platform) [14], specialized for medical text in German. It will be optimized for the use case with historical MDM data from 2011–2021. The information extraction model will be complemented with regular expressions to guarantee high precision. If a parameter cannot be found, the ADBoard should recognize this and check whether a therapy recommendation can still be made at the MDM or if further diagnostics are necessary. Each piece of information automatically extracted and inserted into an empty field will be highlighted as “automatically-inserted data” and appended with its source document and context information. This allows physicians to quickly check the validity of the provided information.

Next, using the complete set of patient data, appropriate treatment options will be recommended. Building on the existing patient health prediction model EffiCare [15], the decision support system will be trained on both structured and unstructured retrospective MDM data. The system will integrate specified clinical guidelines and combine the output of ML and the physicians to form a joint recommendation. The recommendation will be presented together with a confidence score and a justification (explanation), which describes the relevant factors (features) of the decision based on feature importance and Shapley values. Specifically, the corresponding guideline, the agreement with historical MDM decisions (clustering procedure via distance metrics of the feature vectors, such as influential features or cosine distance), and the patient data relevant for decision-making are detailed. Using feedback from MDM physicians, recommendations can be optimized over time according to clinician preference.

#### Onsite and offsite requirements needed to integrate the AI intervention

The AI components will run on a high-performance computing cluster within the hospital infrastructure. The model will be trained on the MDM patient data of the same hospital within the infrastructure, which will be similar to the real test data during the study. We build upon existing proprietary models and technology and integrate the output into the existing MDM dashboard. To do so, a software interface needs to be defined—to send the model results from the backend to the dashboard. The dashboard will then be extended accordingly.

#### Procedure for data acquisition and selection for the AI intervention

The two pretrained AI components run within Charité infrastructure and have access to the necessary patient information across various hospital departments. As the model will not be able to access the real-time data in the hospital information system, the patient data of patients occurring in the next MDM will be automatically collected within an initial step.

#### Procedure for assessing and handling poor quality or unavailable input data

Availability of data and its quality will have a strong influence on the models. Information extraction models might extract false data and thus negatively affect the performance of our models. This will be addressed by providing the source document and the context in which the extracted information occurs. Conversely, if required data are not present in any of the clinical text sources in the hospital, no information can be extracted. This might have an influence on the decision if a patient can be actually discussed in the MDM. It might have an influence on the treatment recommendation, as less information is available, particularly if the missing information is a crucial input parameter. Either way, the recommendation will be provided with a confidence score. If the decision was based on incomplete information, the confidence score would be lower, indicating insecurity of the model.

## Discussion

AI algorithms have been widely tested in medicine, with medical imaging as a well-known area of its application [16]. There remains great potential for AI to be further integrated into other areas of healthcare and medicine, and our study explores the application of AI in liver cancer care. The complexity of liver tumors and their treatment options necessitate the discussion of patients with liver tumors at MDM-C as an integral part of their treatment plan. From our physicians’ experiences, specialist physicians spend a significant amount of time preparing for and attending these meetings. We identified a medical need here and seek to improve the efficiency of this process using a decision algorithm. The literature has several examples of this, with a number of them focusing on breast tumors [17–19]. To our knowledge, this is the first trial to compare the concordance of MDM-C with an AI-based decision algorithm in producing therapy recommendations in participants with liver tumors. When fully developed, the ADBoard algorithm would produce treatment recommendations for cases that can be clearly classified with all the information necessary for decision-making that are not inferior to that of an MDM-C. Reliable recommendations from the ADBoard save physicians time in discussing straightforward cases, which can be funneled to focusing on more complex cases instead. ADBoard will streamline the MDM process further by reducing residents’ administrative workload through automation of form-filling with data directly from the health information system.

Possible limitations of the study are the single-center design, the focus on liver tumor entities, and the inability of the algorithm to consider decision-making factors that may not be included in the hospital information system, such as subjective physical assessments or psychosocial factors.

Potential practical issues in performing the study are external partners in DFKI obtaining the correct permissions and network access to access the Health Data Platform and planning how the therapy recommendations would be displayed during MDMs.

## Trial status

Recruitment has not started. We will begin recruitment in November 2023 and will complete by July 2025.

## Ethics and dissemination

### Research ethics approval

The project was approved by the Ethics Committee of the Charité – Universitätsmedizin Berlin (application number EA4/169/22). Written, informed consent for participation will be obtained from all participants. Participants would potentially join an RCT that assesses the process of MDMs, and this would be explained clearly to them during the process of obtaining informed consent. The model consent form and participation information materials in the German language are available from the corresponding author on request.

### Data management

A data protection concept was developed under the advice of the Clinical Trial Office Charité, based on the European Union General Data Protection Regulation.

The full dataset will be accessible to the physicians. The pseudonymized dataset will be accessible to research team members who require it for analysis purposes. It will be password-protected and stored on a server within the study institution. The PI and other study investigators authorized by the PI will safeguard the final trial dataset. Any data required to support the protocol can be supplied on reasonable request and in line with the data security laws of Berlin, Germany.

Identifiable information about potential and enrolled participants will be collected in REDCap and stored on a local server within the institution. The participants’ data will be pseudonymized and the researchers will work with the pseudonymized data. The participants’ data will be kept for up to 10 years after the end of the trial and then deleted.

The various components for data processing and decision-making will be deployed on a Linux server within the study institution. DFKI researchers will develop the models on this server to ensure that no data will leave the study institution.

To ensure that all collected health data are unidentifiable, the research-related paper forms (e.g., consent forms) and electronic data will be stored physically separately from participants’ medical records. The paper forms are stored in a lockable room in a lockable cupboard accessible only by the principal investigator (PI) and persons authorized by him. The PI is responsible for storing and managing the identification list. The password-protected access authorization to the servers used is exclusively granted by the PI.

### Auditing

The Trial Management Committee and Experimental Surgery Berlin, a research group of the Charité independent of the study research team, meet every 2 months to review trial conduct.

### Plan for dissemination

We intend to disseminate the results of the study to the general medical community via publication in medical journals, regardless of the magnitude or direction of effect. The trial register at ClinicalTrials.gov will be updated with the results as they become available. At the time of manuscript submission, there are no known restrictions to the right to publish study results on any members of the research team.

Subject to German data security law, the full protocol, participant-level dataset, and statistical code are available from the corresponding author on reasonable request, for cooperation purposes only.

### Protocol amendments

Any protocol amendments that may affect the way the study is conducted or its participants, such as changes in study objectives or design, inclusion or exclusion criteria, or outcomes evaluated, will require a formal amendment to the protocol. All members of the research group and the ethics committee must agree upon such amendments before their implementation. Administrative changes that do not have a similar effect are considered minor amendments and will be documented in an addendum to the protocol. All approved protocol amendments will be clearly stated in trial reports.

### Supplementary Information

**Additional file 1.**

## Abbreviations

MDM: Multidisciplinary team meeting
HCC: Hepatocellular carcinoma
GTDS: Gießen Tumor Documentation System
ADBoard: Therapeutic Assistance and Decision algorithms for hepatobiliary tumor Boards
AI: Artificial intelligence
NLP: Natural language processing
ML: Machine learning
MDM-C: Conventional MDM
MDM-AB: ADBoard-supported MDM
REDCap: Research Electronic Data Capture
SCS: System Causability Scale
mEx: Medical information Extraction platform
